# Educational chatbots for project-based learning: investigating learning outcomes for a team-based design course

**DOI:** 10.1186/s41239-021-00302-w

**Published:** 2021-12-15

**Authors:** Jeya Amantha Kumar

**Affiliations:** Centre for Instructional Technology and Multimedia, Universiti Sains Malaysia, Minden, Pulau Pinang Malaysia

**Keywords:** Chatbot, Design education, Teamwork, Project-based learning, Collaborative learning, Mobile learning, Telegram

## Abstract

Educational chatbots (ECs) are chatbots designed for pedagogical purposes and are viewed as an Internet of Things (IoT) interface that could revolutionize teaching and learning. These chatbots are strategized to provide personalized learning through the concept of a virtual assistant that replicates humanized conversation. Nevertheless, in the education paradigm, ECs are still novel with challenges in facilitating, deploying, designing, and integrating it as an effective pedagogical tool across multiple fields, and one such area is project-based learning. Therefore, the present study investigates how integrating ECs to facilitate team-based projects for a design course could influence learning outcomes. Based on a mixed-method quasi-experimental approach, ECs were found to improve learning performance and teamwork with a practical impact. Moreover, it was found that ECs facilitated collaboration among team members that indirectly influenced their ability to perform as a team. Nevertheless, affective-motivational learning outcomes such as perception of learning, need for cognition, motivation, and creative self-efficacy were not influenced by ECs. Henceforth, this study aims to add to the current body of knowledge on the design and development of EC by introducing a new collective design strategy and its pedagogical and practical implications.

## Introduction

Chatbots are defined as computer programs that replicate human-like conversations by using natural language structures (Garcia Brustenga et al., 2018; Pham et al., 2018) in the form of text messages (websites or mobile applications), voice-based (Alexa or Siri), or a combination of both (Pereira et al., 2019; Sandoval, 2018). These automated conversational agents (Riel, 2020) have been significantly used to replicate customer service interaction (Holotescu, 2016) in various domains (Khan et al., 2019; Wang et al., 2021) to an extent it has become a common trend (Wang et al., 2021). The use of chatbots are further expanded due to the affordance, cost (Chocarro et al., 2021), development options (Sreelakshmi et al., 2019; Wang et al., 2021), and adaption facilitated by social network and mobile instant messaging (MIM) applications (apps) (Brandtzaeg & Følstad, 2018; Cunningham-Nelson et al., 2019) such as WhatsApp, Line, Facebook, and Telegrams.

Accordingly, chatbots popularized by social media and MIM applications have been widely accepted (Rahman et al., 2018; Smutny & Schreiberova, 2020) and referred to as mobile-based chatbots. These bots have been found to facilitates collaborative learning (Schmulian & Coetzee, 2019), multimodal communication (Haristiani et al., 2019), scaffolding, real-time feedback (Gonda et al., 2019), personalized learning (Oke & Fernandes, 2020; Verleger & Pembridge, 2019), scalability, interactivity (Dekker et al., 2020) and fosters knowledge creation and dissipation effectively (Verleger & Pembridge, 2019). Nevertheless, given the possibilities of MIM in conceptualizing an ideal learning environment, we often overlook if instructors are capable of engaging in high-demand learning activities, especially around the clock (Kumar & Silva, 2020). Chatbots can potentially be a solution to such a barrier (Schmulian & Coetzee, 2019), especially by automatically supporting learning communication and interactions (Eeuwen, 2017; Garcia Brustenga et al., 2018) for even a large number of students.

Nevertheless, Wang et al. (2021) claims while the application of chatbots in education are novel, it is also impacted by scarcity. Smutny and Schreiberova (2020), Wang et al. (2021), and Winkler and Söllner (2018) added that the current domain of research in educational chatbots (EC) has been focusing on language learning (Vázquez-Cano et al., 2021), economics, medical education, and programming courses. Henceforth, it is undeniable that the role of EC, while not been widely explored outside these contexts (Schmulian & Coetzee, 2019; Smutny & Schreiberova, 2020) due to being in the introductory stages (Chen et al., 2020), are also constrained with limited pedagogical examples in the educational context (Stathakarou et al., 2020). Nevertheless, while this absence is inevitable, it also provides a potential for exploring innovations in educational technology across disciplines (Wang et al., 2021). Furthermore, according to Tegos et al. (2020), investigation on integration and application of chatbots is still warranted in the real-world educational settings. Therefore, the objective of this study is first to address research gaps based on literature, application, and design and development strategies for EC. Next, by situating the study based on these selected research gaps, the effectiveness of EC is explored for team-based projects in a design course using a quasi-experimental approach.

## Literature review

### Chatbots

The term “chatbot” was derived to represent two main attributes which are “chat” in lieu of the conversational attributes and “bot” short for robot (Chocarro et al., 2021). Chatbots are automated programs designed to execute instructions based on specific inputs (Colace et al., 2018) and provide feedback that replicates natural conversational style (Ischen et al., 2020). According to Adamopoulou and Moussiades (2020), there are six main chatbots parameters that determines design and development consideration:

i. knowledge domain—open and closed domains
ii. services—interpersonal, intrapersonal, and inter-agent chatbots
iii. goals—informative, chat-based, or task-based
iv. input processing and response generation—rule-based model, retrieval-based model, and generative model
v. human aid
vi. build—open-source or closed platforms.

These parameters convey that a chatbot can fulfill numerous communication and interaction functionalities based on needs, platforms, and technologies. Typically, they are an exemplary use of artificial intelligence (AI) which conversely initiated various state-of-the-art platforms for developing chatbots such as Google’s DialogFlow, IBM Watson Conversation, Amazon Lex, Flow XO, and Chatterbot (Adamopoulou & Moussiades, 2020). However, while using AI is impressive, chatbots application is limited as it primarily uses the concept of artificial narrow intelligence (ANI) (Holotescu, 2016). Therefore, it can only perform a single task based on a programmed response, such as examining inputs, providing information, and predicting subsequent moves. While limited, ANI is the only form of AI that humanity has achieved to date (Schmulian & Coetzee, 2019). Conversely, such limitation also enables a non-technical person to design and develop chatbots without much knowledge of AI, machine learning, or neuro-linguistic programming (Gonda et al., 2019). While this creates an “openness with IT” (Schlagwein et al., 2017) across various disciplines, big-tech giants such as Google, Facebook, and Microsoft also view chatbots as the next popular technology for the IoT era (Følstad & Brandtzaeg, 2017). Henceforth, if chatbots are able to gain uptake, it will change how people obtain information, communicate (Følstad et al., 2019), learn and gather information (Wang et al., 2021); hence the introduction of chatbots for education.

### Chatbots in education

Chatbots deployed through MIM applications are simplistic bots known as messenger bots (Schmulian & Coetzee, 2019). These platforms, such as Facebook, WhatsApp, and Telegram, have largely introduced chatbots to facilitate automatic around-the-clock interaction and communication, primarily focusing on the service industries. Even though MIM applications were not intended for pedagogical use, but due to affordance and their undemanding role in facilitating communication, they have established themselves as a learning platform (Kumar et al., 2020; Pereira et al., 2019). Henceforth, as teaching is an act of imparting knowledge through effective communication, the ubiquitous format of a mobile-based chatbot could also potentially enhance the learning experience (Vázquez-Cano et al., (2021); thus, chatbots strategized for educational purposes are described as educational chatbots.

Bii (2013) defined educational chatbots as chatbots conceived for explicit learning objectives, whereas Riel (2020) defined it as a program that aids in achieving educational and pedagogical goals but within the parameters of a traditional chatbot. Empirical studies have positioned ECs as a personalized teaching assistant or learning partner (Chen et al., 2020; Garcia Brustenga et al., 2018) that provides scaffolding (Tutor Support) through practice activities (Garcia Brustenga et al., 2018). They also support personalized learning, multimodal content (Schmulian & Coetzee, 2019), and instant interaction without time limits (Chocarro et al., 2021). All the same, numerous benefits have been reported reflecting positive experiences (Ismail & Ade-Ibijola, 2019; Schmulian & Coetzee, 2019) that improved learning confidence (Chen et al., 2020), motivation, self-efficacy, learner control (Winkler & Söllner, 2018), engagement (Sreelakshmi et al., 2019), knowledge retention (Cunningham-Nelson et al., 2019) and access of information (Stathakarou et al., 2020). Furthermore, ECs were found to provide value and learning choices (Yin et al., 2021), which in return is beneficial in customizing learning preferences (Tamayo et al., 2020).

Besides, as ECs promotes anytime anywhere learning strategies (Chen et al., 2020; Ondas et al., 2019), it is individually scalable (Chocarro et al., 2021; Stathakarou et al., 2020) to support learning management (Colace et al., 2018) and delivery of context-sensitive information (Yin et al., 2021). Henceforth, encouraging participation (Tamayo et al., (2020); Verleger & Pembridge, 2019) and disclosure (Brandtzaeg & Følstad, 2018; Ischen et al., 2020; Wang et al., 2021) of personal aspects that were not possible in a traditional classroom or face to face interaction. Conversely, it may provide an opportunity to promote mental health (Dekker et al., 2020) as it can be reflected as a ‘safe’ environment to make mistakes and learn (Winkler & Söllner, 2018). Furthermore, ECs can be operated to answer FAQs automatically, manage online assessments (Colace et al., 2018; Sandoval, 2018), and support peer-to-peer assessment (Pereira et al., 2019).

Moreover, according to Cunningham-Nelson et al. (2019), one of the key benefits of EC is that it can support a large number of users simultaneously, which is undeniably an added advantage as it reduces instructors' workload. Colace et al. (2018) describe ECs as instrumental when dealing with multiple students, especially testing behavior, keeping track of progress, and assigning tasks. Furthermore, ECs were also found to increase autonomous learning skills and tend to reduce the need for face-to-face interaction between instructors and students (Kumar & Silva, 2020; Yin et al., 2021). Conversely, this is an added advantage for online learning during the onset of the pandemic. Likewise, ECs can also be used purely for administrative purposes, such as delivering notices, reminders, notifications, and data management support (Chocarro et al., 2021). Moreover, it can be a platform to provide standard information such as rubrics, learning resources, and contents (Cunningham-Nelson et al., 2019). According to Meyer von Wolff et al (2020), chatbots are a suitable instructional tool for higher education and student are acceptive towards its application.

Conversely, Garcia Brustenga et al. (2018) categorized ECs based on eight tasks in the educational context as described in Table 1. Correspondingly, these tasks reflect that ECs may be potentially beneficial in fulfilling the three learning domains by providing a platform for information retrieval, emotional and motivational support, and skills development.

**Table 1:** Educational task of ECs

Task	Description
Administrative and management	EC is used to aid the onboarding of learning activities
FAQ Platform	EC provides feedback on FAQs for administration or educational topics
Mentoring	EC is used to monitor students learning outcomes cognitively and affectively
Motivational	EC provides emotional and motivational support
Practice specific skills and abilities	EC is used as a practice buddy to learn a language, communication, and programming
Simulations	EC is used to simulate conditions that aid with rehabilitation, such as in healthcare
Reflection and metacognitive strategies	EC is used as a skillful classmate that aids learning
Student learning assessment	ECs measure learning outcomes quickly and routinely

Albeit, from the instructor’s perspective, ECs could be intricate and demanding, especially when they do not know to code (Schmulian & Coetzee, 2019); automation of some of these interactions could benefit educators in focusing on other pedagogical needs (Gonda et al., 2019). Nevertheless, enhancing such skills is often time-consuming, and teachers are usually not mentally prepared to take up a designer's (Kim, 2021) or programmer's role. The solution may be situated in developing code-free chatbots (Luo & Gonda, 2019), especially via MIM (Smutny & Schreiberova, 2020).

By so, for EC development, it is imperative to ensure there are design principles or models that can be adapted for pedagogical needs. At the same time, numerous models have been applied in the educational context, such as CommonKADS (Cameron et al., 2018), Goal-Oriented Requirements Engineering (GORE) (Arruda et al., 2019), and retrieval-based and QANet models (Wu et al., 2020). Nevertheless, these models reflect a coding approach that does not emphasize strategies or principles focusing on achieving learning goals. While Garcia Brustenga et al. (2018), Gonda et al. (2019), Kerly et al. (2007), Satow (2017), Smutny and Schreiberova (2020), and Stathakarou et al. (2020) have highlighted some design guidelines for EC, imperatively a concise model was required. Therefore, based on the suggestions of these empirical studies, the researcher identified three main design attributes: reliability, pedagogy, and experience (Table 2).

**Table 2:** EC design strategies from empirical findings

Factors	Contextualization of RiPE	Kerly et al. (2007)	Gonda et al. (2019)	Smutny and Schreiberova (2020)	Satow (2017)	Stathakarou et al. (2020)	Garcia Brustenga et al. (2018)
Reliability	Easy access to a stable platform	√		√		√	
	Privacy	√					
	Feedback for continuous improvement	√					
Pedagogy	Learning contents that are reliable, precise, and specific to the subject		√	√	√	√	√
	Alignment with learning goals	√	√		√		√
	Active learning that promotes reflection and metacognition	√	√				
	Encourages communication and collaboration in the learning community		√				
	Personalize learning and feedbacks		√		√		
	Progress management		√	√			
Experience	Realistic humanized communication with social cue	√		√		√	
	Affective interaction (greetings, humor, anthropomorphism, empathy)			√	√	√	√
	Social media and MIM affordance					√	

Nevertheless, it was observed that the communicative aspect was absent. Undeniably, chatbots are communication tools that stimulate interpersonal communication (Ischen et al., 2020; Wang et al., 2021); therefore, integrating interpersonal communication was deemed essential. Interpersonal communication is defined as communication between two individuals who have established a relationship (Devito, 2018), and such a relationship is also significant through MIM to represent the communication between peers and instructors (Chan et al., 2020). Furthermore, according to Han and Xu (2020), interpersonal communication moderates the relationship and perception that influences the use of an online learning environment. According to Hobert and Berens (2020), while chatbot interaction could facilitate small talk that could influence learning, such capabilities should not be overemphasize. Therefore, it was concluded that four fundamental attributes or strategies were deemed critical for EC design: Reliability, *i*nterpersonal communication, Pedagogy, and *E*xperience (RiPE), which are explained in Table 3.

**Table 3:** Describing RiPE for educational chatbots

Factors	Description
Reliability	The chatbot should be easy to access through a stable and private platform where the learner can depend on the chatbot to gain continuous feedback with confidence
Interpersonal communication	The chatbot should establish a relationship between learner-learner and learner-instructor through activities that enable them to relate, share information, communicate, and/or collaborate
Pedagogy	The chatbot provides learning content and activities that align with the learning goals of the course. Therefore, while facilitating a personalized learning platform, the chatbot should also embody active learning and communication strategies that allow the instructor to monitor learning progress
Experience	The chatbot should be deployed on a preferred communication platform and reflect how learners communicate in a natural online setting. Affective interaction such as greetings, humor, emojis, and/or empathy should also be included to improve emotional engagement. Furthermore, the interaction should be based on small learning units strategized for micro-learning

Nevertheless, ECs are not without flaws (Fryer et al., 2019). According to Kumar and Silva (2020), acceptance, facilities, and skills are still are a significant challenge to students and instructors. Similarly, designing and adapting chatbots into existing learning systems is often taxing (Luo & Gonda, 2019) as instructors sometimes have limited competencies and strategic options in fulfilling EC pedagogical needs (Sandoval, 2018). Moreover, the complexity of designing and capturing all scenarios of how a user might engage with a chatbot also creates frustrations in interaction as expectations may not always be met for both parties (Brandtzaeg & Følstad, 2018). Hence, while ECs as conversational agents may have been projected to substitute learning platforms in the future (Følstad & Brandtzaeg, 2017), much is still to be explored from stakeholders' viewpoint in facilitating such intervention.

### Research gaps in EC research

Three categories of research gaps were identified from empirical findings (i) learning outcomes, (ii) design issues, and (iii) assessment and testing issues. Firstly, research gaps concerning learning outcomes are such as measuring effectiveness (Schmulian & Coetzee, 2019), perception, social influence (Chaves & Gerosa, 2021), personality traits, affective outcomes (Ciechanowski et al., 2019; Winkler & Söllner, 2018), acceptance (Chen et al., 2020; Chocarro et al., 2021), satisfaction (Stathakarou et al., 2020), interest (Fryer et al., 2019), motivation, learning performance (Yin et al., 2021), mental health (Brandtzaeg & Følstad, 2018), engagement (Riel, 2020) and cognitive effort (Nguyen & Sidorova, 2018). EC studies have primarily focused on language learning, programming, and health courses, implying that EC application and the investigation of learning outcomes have not been investigated in various educational domains and levels of education.

Next, as for design and implementation issues, a need to consider strategies that align ECs application for teaching and learning (Haristiani et al., 2019; Sjöström et al., 2018) mainly to supplement activities that can be used to replace face-to-face interactions (Schmulian & Coetzee, 2019) has been implied. According to Schmulian and Coetzee (2019), there is still scarcity in mobile-based chatbot application in the educational domain, and while ECs in MIM has been gaining momentum, it has not instigated studies to address its implementation. Furthermore, there are also limited studies in strategies that can be used to improvise ECs role as an engaging pedagogical communication agent (Chaves & Gerosa, 2021). Besides, it was stipulated that students' expectations and the current reality of simplistic bots may not be aligned as Miller (2016) claims that ANI’s limitation has delimited chatbots towards a simplistic menu prompt interaction.

Lastly, in regards to assessment issues, measurement strategies for both intrinsic and extrinsic learning outcomes (Sjöström et al., 2018) by applying experimental approaches to evaluate user experience (Fryer et al., 2019; Ren et al., 2019) and psychophysiological reactions (Ciechanowski et al., 2019) has been lacking. Nevertheless, Hobert (2019) claims that the main issue with EC assessment is the narrow view used to evaluate outcomes based on specific fields rather than a multidisciplinary approach. Moreover, evaluating the effectiveness of ECs is a complex process (Winkler & Söllner, 2018) as it is unclear what are the characteristics that are important in designing a specific chatbot (Chaves & Gerosa, 2021) and how the stakeholders will adapt to its application to support teaching and learning (Garcia Brustenga et al., 2018). Furthermore, there is a need for understanding how users experience chatbots (Brandtzaeg & Følstad, 2018), especially when they are not familiar with such intervention (Smutny & Schreiberova, 2020). Conversely, due to the novelty of ECs, the author has not found any studies pertaining to ECs in design education, project-based learning, and focusing on teamwork outcomes.

### Purpose of the study

This study aims to investigate the effects of ECs for an Instructional Design course that applies team-based project towards learning outcomes, namely learning performance, perception of learning, need for cognition, motivation, creative self-efficacy, and teamwork. Learning performance is defined as the students' combined scores accumulated from the project-based learning activities in this study. Next, perception of the learning process is described as perceived benefits obtained from the course (Wei & Chou, 2020) and the need for cognition as an individual’s tendency to participate and take pleasure in cognitive activities (de Holanda Coelho et al., 2020). The need for cognition also indicates positive acceptance towards problem-solving (Cacioppo et al., 1996), enjoyment (Park et al., 2008), and it is critical for teamwork, as it fosters team performance and information-processing motivation (Kearney et al., 2009). Henceforth, we speculated that EC might influence the need for cognition as it aids in simplifying learning tasks (Ciechanowski et al., 2019), especially for teamwork.

Subsequently, motivational beliefs are reflected by perceived self-efficacy and intrinsic values students have towards their cognitive engagement and academic performance (Pintrich & de Groot, 1990). According to Pintrich et al. (1993), self-efficacy and intrinsic value strongly correlate with task value (Eccles & Wigfield, 2002), such as interest, enjoyment, and usefulness. Furthermore, Walker and Greene (2009) explain that motivational factors that facilitate learning are not always solely reliant on self-efficacy, and Pintrich and de Groot (1990) claims that a combination of self-efficacy and intrinsic value is better in explaining the extent to which students are willing to take on the learning task. Ensuing, the researcher also considered creative self-efficacy, defined as the students' belief in producing creative outcomes (Brockhus et al., 2014). Prior research has not mentioned creativity as a learning outcome in EC studies. However, according to Pan et al. (2020), there is a positive relationship between creativity and the need for cognition as it also reflects individual innovation behavior. Likewise, it was deemed necessary due to the nature of the project, which involves design. Lastly, teamwork perception was defined as students' perception of how well they performed as a team to achieve their learning goals. According to Hadjielias et al. (2021), the cognitive state of teams involved in digital innovations is usually affected by the task involved within the innovation stages. Hence, the consideration of these variables is warranted.

Therefore, it was hypothesized that using ECs could improve learning outcomes, and a quasi-experimental design comparing EC and traditional (CT) groups were facilitated, as suggested by Wang et al. (2021), to answer the following research questions.

i. Does the EC group perform better than students who learn in a traditional classroom setting?
ii. Do students who learn with EC have a better perception of learning, need for cognition, motivational belief, and creative self-efficacy than students in a traditional classroom setting?
iii. Does EC improve teamwork perception in comparison to students in a traditional classroom setting?

### Educational chatbot design, development, and deployment

According to Adamopoulou and Moussiades (2020), it is impossible to categorize chatbots due to their diversity; nevertheless, specific attributes can be predetermined to guide design and development goals. For example, in this study, the rule-based approach using the if-else technique (Khan et al., 2019) was applied to design the EC. The rule-based chatbot only responds to the rules and keywords programmed (Sandoval, 2018), and therefore designing EC needs anticipation on what the students may inquire about (Chete & Daudu, 2020). Furthermore, a designer should also consider chatbot's capabilities for natural language conversation and how it can aid instructors, especially in repetitive and low cognitive level tasks such as answering FAQs (Garcia Brustenga et al., 2018). As mentioned previously, the goal can be purely administrative (Chocarro et al., 2021) or pedagogical (Sandoval, 2018).

Next, as for the design and development of the EC, *Textit* (https://textit.com/), an interactive chatbots development platform, was utilized. Textit is a third-party software developed by Nyaruka and UNICEF that offers chatbots building possibilities without coding but using the concept of flows and deployment through various platforms such as Facebook Messenger, Twitter, Telegram, and SMS. For the design of this EC, Telegram was used due to data encryption security (de Oliveira et al., 2016), cloud storage, and the privacy the student and instructor would have without using their personal social media platforms. Telegram has been previously used in this context for retrieving learning contents (Rahayu et al., 2018; Thirumalai et al., 2019), information and progress (Heryandi, 2020; Setiaji & Paputungan, 2018), learning assessment (Pereira, 2016), project-based learning, teamwork (Conde et al., 2021) and peer to peer assessment (P2P) (Pereira et al., 2019).

Subsequently, the chatbot named after the course code (QMT212) was designed as a teaching assistant for an instructional design course. It was targeted to be used as a task-oriented (Yin et al., 2021), content curating, and long-term EC (10 weeks) (Følstad et al., 2019). Students worked in a group of five during the ten weeks, and the ECs' interactions were diversified to aid teamwork activities used to register group members, information sharing, progress monitoring, and peer-to-peer feedback. According to Garcia Brustenga et al. (2018), EC can be designed without educational intentionality where it is used purely for administrative purposes to guide and support learning. Henceforth, 10 ECs (Table 4) were deployed throughout the semester, where EC1-EC4 was used for administrative purposes as suggested by Chocarro et al. (2021), EC5-EC6 for assignment (Sjöström et al., 2018), EC7 for user feedback (Kerly et al., 2007) and acceptance (Yin et al., 2021), EC8 for monitoring teamwork progress (Colace et al., 2018), EC9 as a project guide FAQ (Sandoval, 2018) and lastly EC10 for peer to peer assessment (Colace et al., 2018; Pereira et al., 2019). The ECs were also developed based on micro-learning strategies to ensure that the students do not spend long hours with the EC, which may cause cognitive fatigue (Yin et al., 2021). Furthermore, the goal of each EC was to facilitate group work collaboration around a project-based activity where the students are required to design and develop an e-learning tool, write a report, and present their outcomes. Next, based on the new design principles synthesized by the researcher, RiPE was contextualized as described in Table 5.

**Table 4:** Description of ECs and objectives

EC	Name of Bot	Objective	Respondents
1	Welcome Bot	i.To gather student information such as full name, nickname, gender, student ID, email, and Telegram ID Number ii.To update the user database iii.To obtain permission to be contacted through Telegram and to use their data based on the data protection act	All
2	Group Registration Bot	To group students and update the user database	All
3	Group leader registration Bot	To register as a group leader or team member and update the user database	All
4	Project registration Bot	To update project name and acronym	Group leader
5	Assignment Bot	To access and download learning contents	All
6	Picaso Bot	To upload contents through the bot	All
7	Perception bot	To gather feedback on the EC and acceptance	All
8	Progress Bot	To rate students' self-perceived progress in their project, identify teamwork and project development issues	All
9	Report writing Bot	FAQ to guide students on how to write the project report	All
10	Peer to peer evaluation Bot	Peer to peer feedback platform for presentation	All

**Table 5:** Conceptualization of RiPE in the design of the EC

RiPE	Contextualization in the EC
Reliability	MIM using Telegram provides easy access and has added privacy features of the data shared. EC1 was also designed to gather feedback on the privacy and confidentiality agreement using students' data and Telegram account details. Example: "Next, we understand that using SMS is going to be expensive for you. Hence, we choose to use Telegram as you will be able to use any WIFI system or data available. Telegram cloud services and enhanced security system will also provide privacy and confidentiality" "So, @results.nick_name, do you agree to use Telegram as a method for contacting you? All your data will be private and confidential and be used only for this study. None of your personal details will be made public in term of knowledge dissemination 1 = Yes, 2 = No"
	Next, feedback for continuous improvement was established in EC7 on acceptance. Example interaction: “Well, that's interesting…. what don’t you like about it?”
	The EC also allows users to come back to any interaction by using keywords, example interaction: "Well, I guess you are busy so anytime you need my help just type "/report" and I will be here to help you"
*i*nterpersonal communication	The ECs were designed to mimic interpersonal communication. Example interactions are: “Ohh…that's too bad. What do you think are the issues you are facing?” “I would like to help you better. Do you see issues in teamwork?” “Hi there @contact.first_name, I noticed you didn't answer the questions yet. I (QMT212 Bot) have been contacting you for a bit now. So, I would like to know how you feel about me and your overall perception of using Telegrams Bots for learning.”
	The communications were also personalized by using nicknames (@results.nick_name) and first names (@contact.first_name)
Learning	Learning contents were designed to be specific to the subject. Example interactions: "Some example subtopic topics that should be added are: i. Gagnes 9 Events of Instructions ii. Instructional Strategies iii. Formative Assessment Would you like to have more information about these subtopics?"
	Similarly, active learning promoting reflection, metacognition, and communication. Example:
	“How do you feel about the prototype product your group has developed to date?”
	The EC also enables students to receive notification, contents, and guidelines, examples are: "Hi @contact.first_name, it is time to complete Assignment 1. This is a group work where you are asked to describe your product's initial phase (proposal). You will have to integrate what you have studied to date into this proposal" "That's great. Next, these videos may be helpful in understanding how to complete the assignment. The deadline for your submission will be on @ date.assignment1. Good Luck!!!"
	The EC was also used as a platform for peer-to-peer assessment where the feedback was privately delivered through email to the respective individuals confidentially. Example interaction: "Hi @contact.first_name, thank you for deciding to rate your friends. Your feedback is confidential and will be emailed personally to the group members for improvement"
*E*xperience	Realistic humanized communication with affective interaction through greeting using users name and nickname, inspirational quotes and empathy were also designed such as: "Find a group of people who challenge and inspire you, spend a lot of time with them, and it will change your life."—Amy Poehler “Thank you. I am sure you will do an excellent job @contact.first_name!!” “Thank you @results.nick_name. As you know I am a bot, and I can't identify if you are a Male/ Female by your name. Therefore, can you let me know your gender? 1: Male 2: Female” "Interesting……..That looks like a beautiful artwork Picasso !!!!" "Do you know that you can use Autodraw to design a unique logo for your products?"

**Fig. 1:**
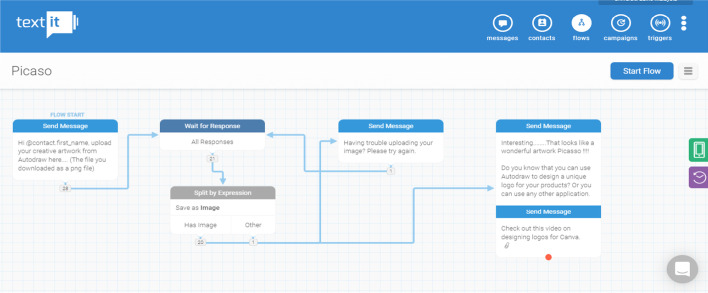
Textit flow diagrams

**Fig. 2:**
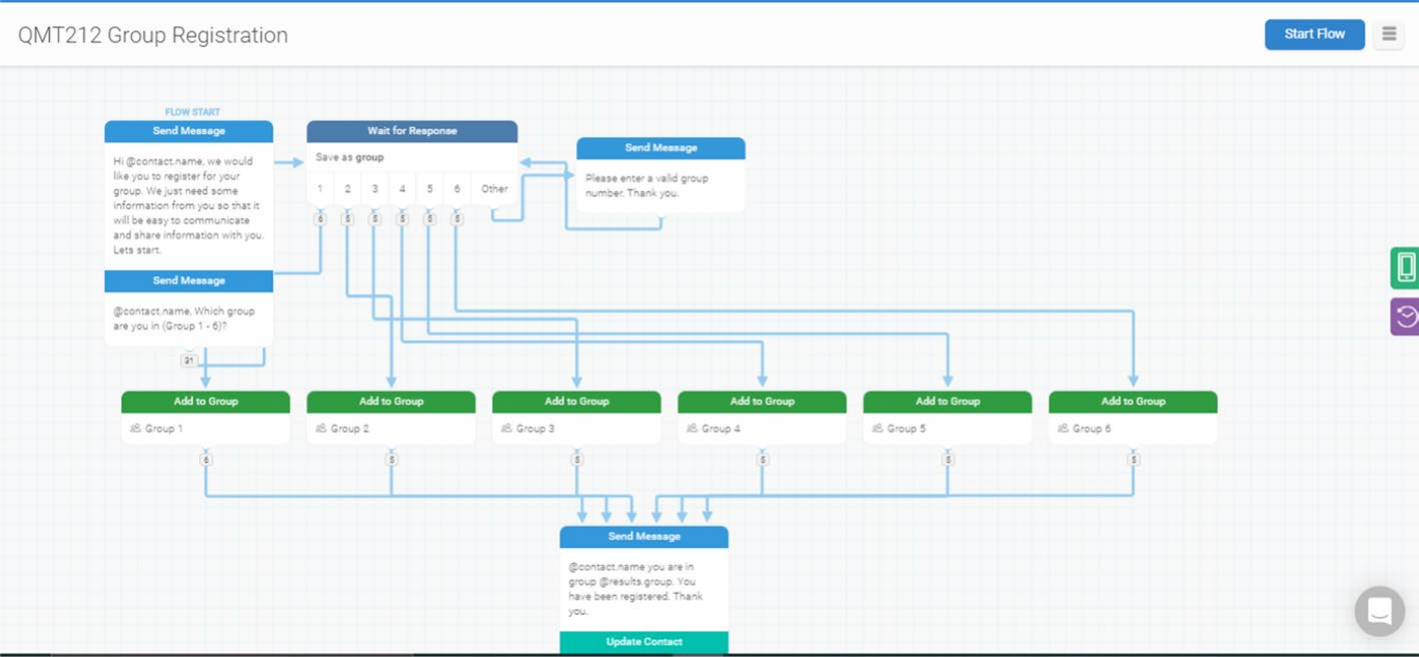
Textit flow diagram for group registration

**Fig. 3:**
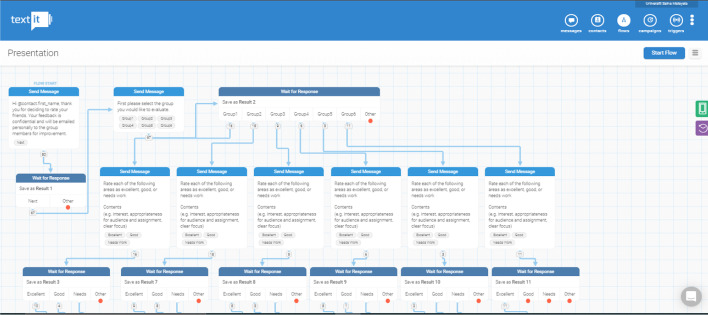
Textit flow diagram for peer to peer evaluation

**Fig. 4:**
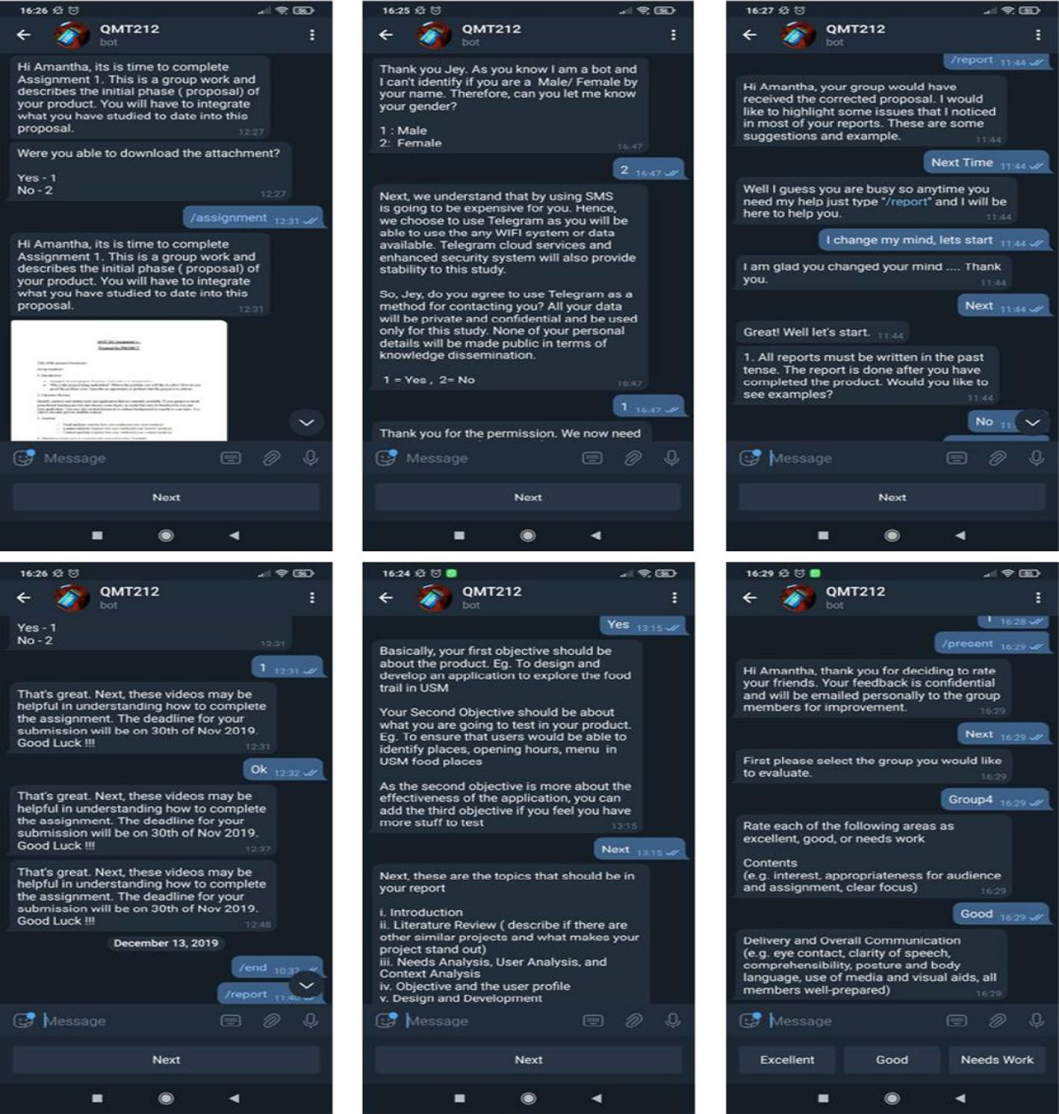
Telegram screenshots of the EC

Example flow diagrams from Textit for the design and development of the chatbot are represented in Fig. 1. The number of choices and possible outputs determine the complexity of the chatbot where some chatbots may have simple interaction that requires them to register their groups (Fig. 2) or much more complex interaction for peer-to-peer assessment (Fig. 3). Example screenshots from Telegram are depicted in Fig. 4.

## Methodology

### Participants

The participants of this study were second-year Bachelor of Education (Teaching English to Speakers of Other Languages (TESOL)) who are minoring in multimedia and currently enrolled in a higher learning institute in Malaysia. The 60 students were grouped into two classes (30 students per class) as either traditional learning class (control group-CT) or chatbot learning class (treatment group-EC). Out of the 60 participants, only 11 were male, 49 were female, and such distribution is typical for this learning program. Both groups were exposed to the same learning contents, class duration, and instructor, where the difference is only denoted towards different class schedules, and only the treatment group was exposed to EC as an aid for teaching and learning the course. Both groups provided written consent to participate in the study and were given honorarium for participation. However, additional consent was obtained from the EC group in regards of data protection act as the intervention includes the use of social media application and this was obtained through EC1: Welcome Bot.      

### The course

The instructional design course aims to provide fundamental skills in designing effective multimedia instructional materials and covers topics such as need analysis, instructional analysis, learner analysis, context analysis, defining goals and objectives, developing instructional strategy and materials, developing assessment methods, and assessing them by conducting formative and summative assessments. The teaching and learning in both classes are identical, wherein the students are required to design and develop a multimedia-based instructional tool that is deemed their course project. Students independently choose their group mates and work as a group to fulfill their project tasks. Moreover, both classes were also managed through the institution's learning management system to distribute notes, attendance, and submission of assignments.

### Procedure

**Fig. 5:**
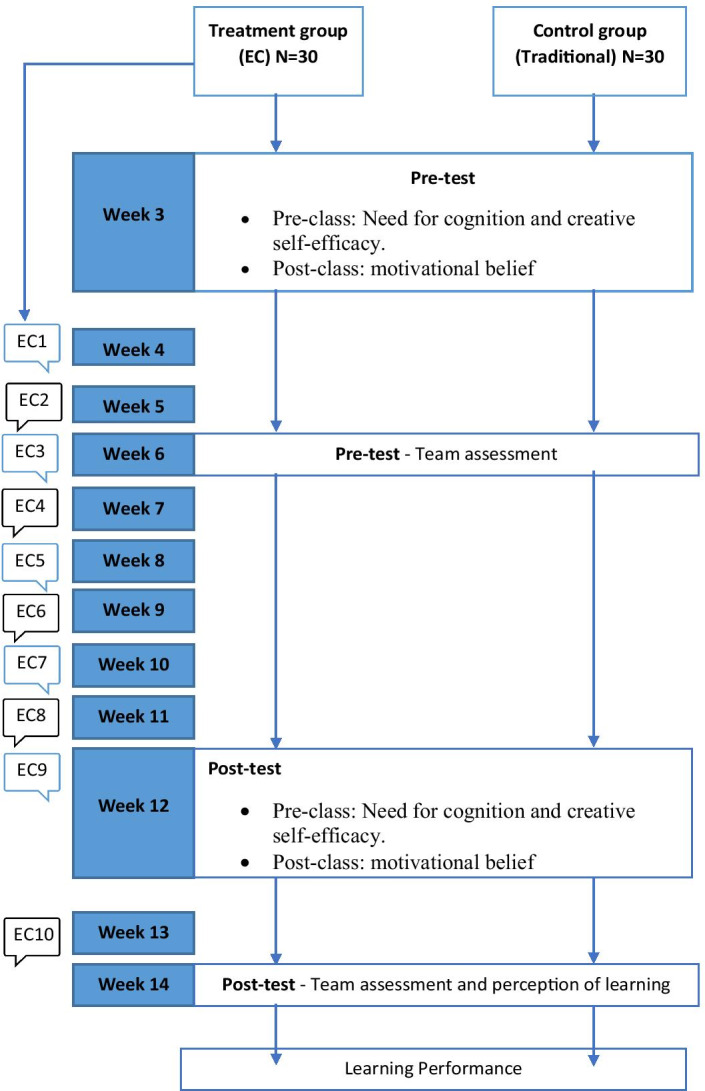
Study procedure

This study applies an interventional study using a quasi-experimental design approach. Creswell (2012) explained that education-based research in most cases requires intact groups, and thus creating artificial groups may disrupt classroom learning. Therefore, one group pretest–posttest design was applied for both groups in measuring learning outcomes, except for learning performance and perception of learning which only used the post-test design. The total intervention time was ten weeks, as represented in Fig. 5. The EC is usually deployed for the treatment class one day before the class except for EC6 and EC10, which were deployed during the class. Such a strategy was used to ensure that the instructor could guide the students the next day if there were any issues.

### Measures

This study integrates five instruments which measure perception of learning (Silva et al., 2017), perceived motivation belief using the Motivated Strategies for Learning Questionnaire (MSLQ) (Pintrich & de Groot, 1990) and modified MSLQ (Silva et al., 2017), need for cognition using the Need for Cognition Scale–6 (NCS-6) (de Holanda Coelho et al., 2020), creative self-efficacy from the Creative Self-Efficacy (QCSE) (Brockhus et al., 2014) and teamwork using a modified version of Team Assessment Survey Questions (Linse, 2007). The teamwork survey had open-ended questions, which are:

i. Give one specific example of something you learned from the team that you probably would not have learned on your own.
ii. Give one specific example of something other team members learned from you that they probably would not have learned without you.
iii. What problems have you had interacting as a team so far?
iv. Suggest one specific, practical change the team could make that would help improve everyone’s learning.

The instruments were rated based on the Likert scale ranging from 1 (strongly disagree) to 5 (strongly agree) and administered using Google Forms for both groups. Where else, learning performance was assessed based on the assessment of the project, which includes report, product, presentation, and peer-to-peer assessment.

A series of one-way analyses of covariance (ANCOVA) was employed to evaluate the difference between the EC and CT groups relating to the need for cognition, motivational belief for learning, creative self-efficacy, and team assessment. As for learning performance, and perception of learning, a t-test was used to identify the difference between the groups. The effect size was evaluated according to Hattie (2015), where an average effect size (Cohen’s *d*) of 0.42 for an intervention using technologies for college students is reflected to improve achievement (Hattie, 2017). Furthermore, as the teamwork has open-ended questions, the difference between the groups was evaluated qualitatively using Text analysis performed using the Voyant tool at https://voyant-tools.org/ (Sinclair & Rockwell, 2021). Voyant tools is an open-source online tool for text analysis and visualization (Hetenyi et al., 2019), and in this study, the collocates graphs were used to represent keywords and terms that occur in close proximity representing a directed network graph.

## Results

### Learning performance for the course

The EC group (µ = 42.500, SD = 2.675) compared the CT group (µ = 39.933, SD = 2.572) demonstrated significant difference at *t* (58) = 3.788, *p* = 0.000, *d* = 0.978; hence indicating difference in learning achievement where the EC group outperformed the control group. The Cohen’s *d* value as described by Hattie (2017) indicated that learning performance improved by the intervention.

### Need for cognition

The initial Levine’s test and normality indicated that the homogeneity of variance assumptions was met at F (1,58) = 0.077, p = 0.782. The adjusted means of µ = 3.416 for the EC group and µ = 3.422 for the CT group indicated that the post-test scores were not significant at F (1, 57) = 0.002, *p* = 0.969, η2p = 0.000, *d* = 0.012); hence indicating that student’s perception of enjoyment and tendency to engage in the course is similar for both groups.

### Motivational beliefs

The initial Levine’s test and normality indicated that the homogeneity of variance assumptions was met at *F* (1,58) = 0.062, p = 0.804. The adjusted means of µ = 4.228 for the EC group and µ = 4.200 for the CT group indicated that the post-test scores were not significant at F (1, 57) = 0.046, *p* = 0.832, η2p = 0.001, *d* = 0.056); hence indicating that the student’s motivation to engage in the course are similar for both groups.

### Creative self-efficacy

The initial Levine’s test and normality indicated that the homogeneity of variance assumptions was met at F (1,58) = 0.808, p = 0.372. The adjusted means of µ = 3.566 for the EC group and µ = 3.627 for the CT group indicated that the post-test scores were not significant at F (1, 57) = 0.256, *p* = 0.615, η2p = 0.004, *d* = 0.133); hence indicating that the student’s perception of creative self-efficacy was similar for both groups.

### Perception of learning

The EC group (µ = 4.370, SD = 0.540) compared the CT group (µ = 4.244, SD = 0.479) demonstrated no significant difference at *t* (58) = 0.956, p = 0.343, *d* = 0.247; hence indicating no difference in how students perceived their learning process quantitively. Nevertheless, we also questioned what impacted their learning (project design and development) the most during the course, and the findings, as shown in Table 6, indicated that both groups (EC = 50.00% and CT = 86.67%) found the group learning activity as having the most impact. The control group was more partial towards the group activities than the EC group indicating online feedback and guidance (30.00%) and interaction with the lecturer as an inequitable influence. It was also indicated in both groups that constructive feedback was mostly obtained from fellow course mates (EC = 56.67%, CT = 50.00%) and the instructor (EC = 36.67%, CT = 43.33%) (Table 7) while minimum effort was made to get feedback outside the learning environment.

**Table 6:** Learning activities impacting project design and development

Groups	Online feedback and guidance	Group activities	Interaction with lecture
EC	30.00% (N = 9)	50.00% (N = 15)	20.00% (N = 6)
CT	0.10% (N = 3)	86.67% (N = 26)	0.06% (N = 2)

**Table 7:** Constructive feedback source

Groups	Group mates	Instructor	Other students	Others (example: industry experts, Web programmer)
EC	56.67% (N = 17)	36.67% (N = 11)	0.06% (N = 2)	0
CT	50.00% (N = 15)	43.33% (N = 13)	0.06% (N = 2)	0

### Team assessment

The initial Levine’s test and normality indicated that the homogeneity of variance assumptions was met at F (1,58) = 3.088, p = 0.051. The adjusted means of µ = 4.518 for the experimental group and µ = 4.049 for the CT group indicated that the post-test scores were significantly different at *F* (1, 57) = 5.950, *p* = 0.018, η2p = 0.095, *d* = 0.641; hence indicating that there was a significant difference between groups in how they performed in teams. The Cohen’s d value, as described by Hattie (2017), indicated that the intervention improved teamwork.

Next, we questioned their perception of teamwork based on what they learned from their teammates, what they felt others learn from them, the problem faced as a team, and recommendations to improve their experience in the course. Based on the feedback, themes such as teamwork, technology, learning management, emotional management, creativity, and none were identified to categories the feedback. The descriptive data are represented in Table 8 for both the groups and the trends reflecting the changes in feedback are described as follow:

**Table 8:** Comparison between EC and CT teamwork perception

	Learn from others				Others learn from you				Problems				Recommendation			
	PRE		Post		PRE		Post		PRE		Post		PRE		Post	
Themes	A	B	A	B	A	B	A	B	A	B	A	B	A	B	A	B
Teamwork	30.00% (N = 9)	40.00% (N = 12)	43.33% (N = 13)	43.33% (N = 13)	13.33% (N = 4)	16.67% (N = 5)	23.33% (N = 7)	16.67% (N = 5)	30.00% (N = 9)	36.67% (N = 11)	50.00% (N = 15)	66.67% (N = 20)	50.00% (N = 15)	90.00% (N = 27)	90.00% (N = 27)	53.33% (N = 16)
Technology	26.67% (N = 8)	30.00% (N = 9)	26.67% (N = 8)	30.00% (N = 9)	3.33% (N = 1)	3.33% (N = 1)	23.33% (N = 7)	16.67% (N = 5)	6.67% (N = 2)	3.33% (N = 1)	6.67% (N = 2)	–	16.67% (N = 5)	3.33% (N = 1)	–	3.33% (N = 1)
Creativity	30.00% (N = 9)	6.67% (N = 2)	10.00% (N = 3)	3.33% (N = 1)	30.00% (N = 9)	10.00% (N = 3)	6.67% (N = 2)	6.67% (N = 2)	13.33% (N = 4)	–	–	–	3.33% (N = 1)	–	–	–
Learning management	10.00% (N = 3)		13.33% (N = 4)	6.67% (N = 2)	10.00% (N = 3)	13.33% (N = 4)	16.67% (N = 5)	23.33% (N = 7)	6.67% (N = 2)	36.67% (N = 11)	6.67% (N = 2)	16.67% (N = 5)	30.00% (N = 9)	3.33% (N = 1)	6.67% (N = 2)	23.33% (N = 7)
Emotional management	3.33% (N = 1)	10.00% (N = 3)	6.67% (N = 2)	13.33% (N = 4)	13.33% (N = 4)	10.00% (N = 3)	16.67% (N = 5)	16.67% (N = 5)	–	–	3.33% (N = 1)	–	–	–	–	10.00% (N = 3)
Others	–	–	–	3.33% (N = 1)	30.00% (N = 9)	46.67% (N = 14)	13.33% (N = 4)	6.67% (N = 2)	10.00% (N = 3)	23.33% (N = 7)	6.67% (N = 2)	16.67% (N = 5)	–	3.33% (N = 1)	3.33% (N = 1)	10.00% (N = 3)

i. Respondent learned from teammates

**Fig. 6:**
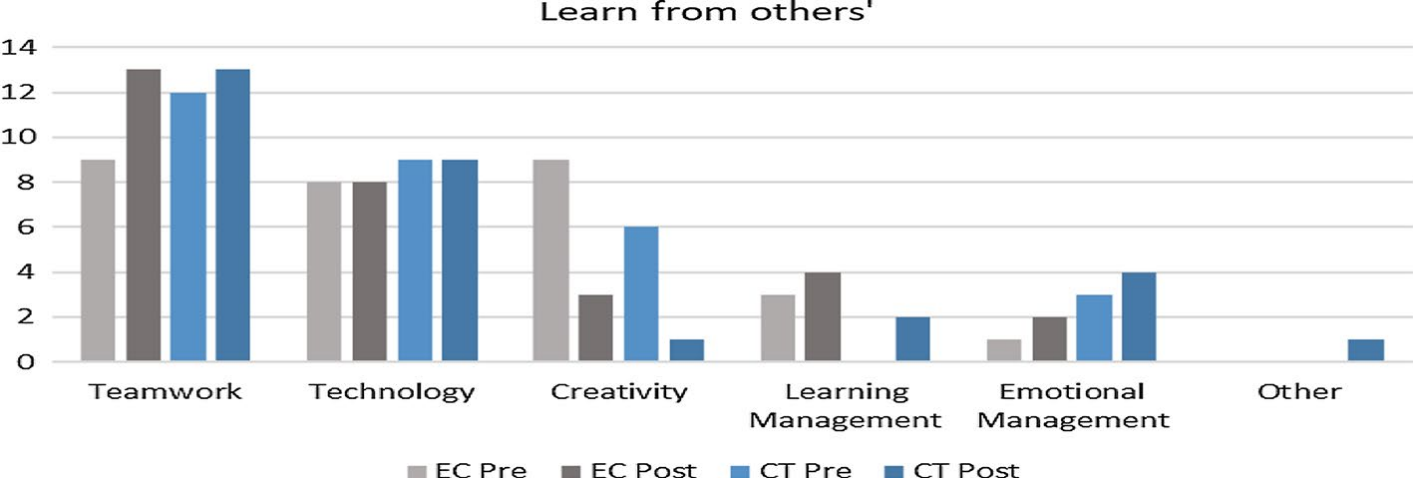
Change in perception pre and post-intervention based on aspects learn from teammates

This question reflects on providing feedback on one aspect they have learned from their team that they probably would not have learned independently. Based on Fig. 6, the illustration describes changes in each group (EC and CT) pre and post-intervention. First, teamwork showed an increasing trend for EC, whereas CT showed slight changes pre and post-intervention.

**Fig. 7:**
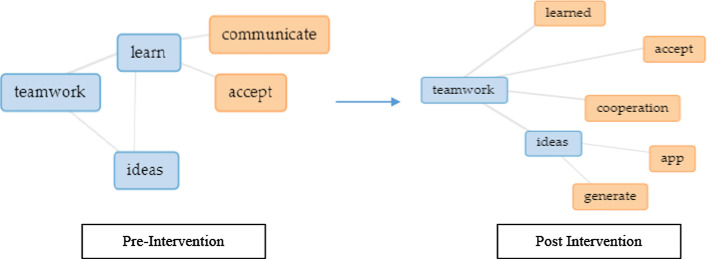
Change in perception for the EC group based on aspects learn from teammate

Next, using text analysis collocates graphs (Fig. 7) for EC post-intervention, a change was observed indicating teamwork perception resonating from just learning new ideas, communicating, and accepting opinions towards a need to cooperate as a team to ensure they achieve their goal of developing the project. It was observed that communicating merely was not the main priority anymore as cooperation towards problem-solving is of utmost importance. Example feedbacks are such as, “I learned teamwork and how to solve complicated problems” and “The project was completed in a shorter period of time, compared to if I had done it by myself.” Next, in both groups, creativity seems to have declined from being an essential aspect in the project's initial phase as it declines towards the end of the semester, whereas an increment was noticed in giving more importance to emotional management when handling matters of the project. Example feedback is such as “I learn to push myself more and commit to the project's success.” Nevertheless, in both groups, all the trends are almost similar.

ii. Teammates learned from the respondent.

**Fig. 8:**
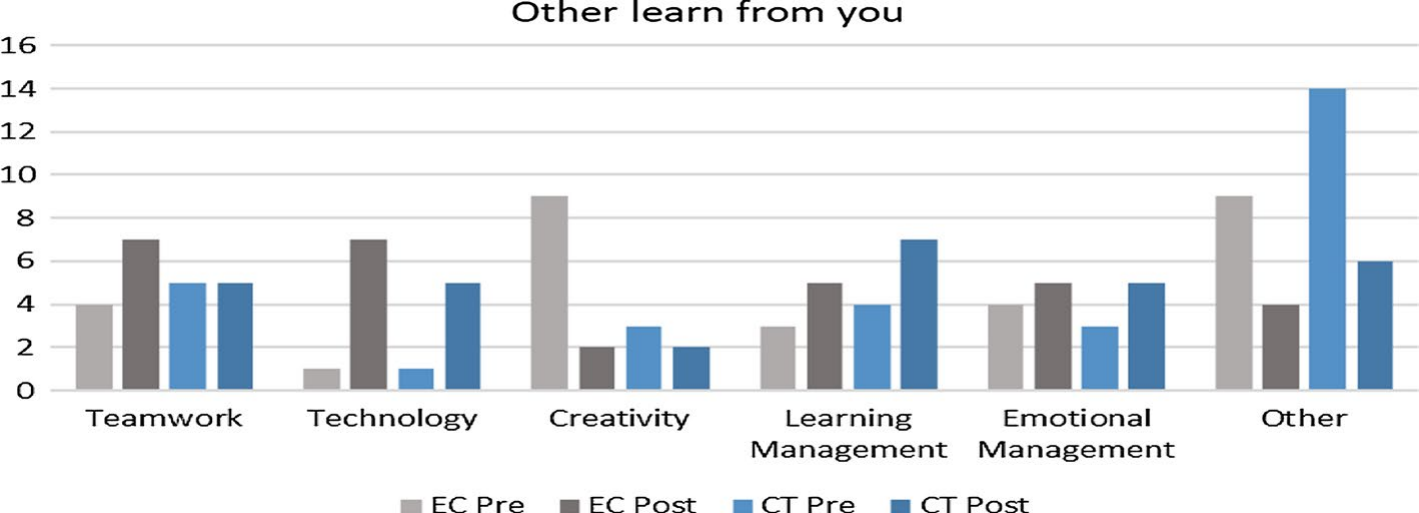
Change in perception pre and post-intervention based on aspects teammates learned from respondents

This question reflects on an aspect the respondent believes that their team members have learned from them. Initially, both groups reported being unaware of their contribution by stating “nothing” or “I don’t know” which was classified as “other” (Fig. 8). Nevertheless, intriguingly both groups showed a decline in such negative perception post-intervention, which can be attributed to self-realization of their contribution in task completion. Furthermore, different trends were observed between both groups for teamwork, where the EC group showed more references to increased teamwork contribution, where else the CT group remained unaffected post-intervention. In terms of technology application, the respondents in both groups described how they were a valuable resource for teaching their peers about technology, where one respondent stated that “My friends learn how to make an application from me.”

iii. Problem respondent faced as a team

**Fig. 9:**
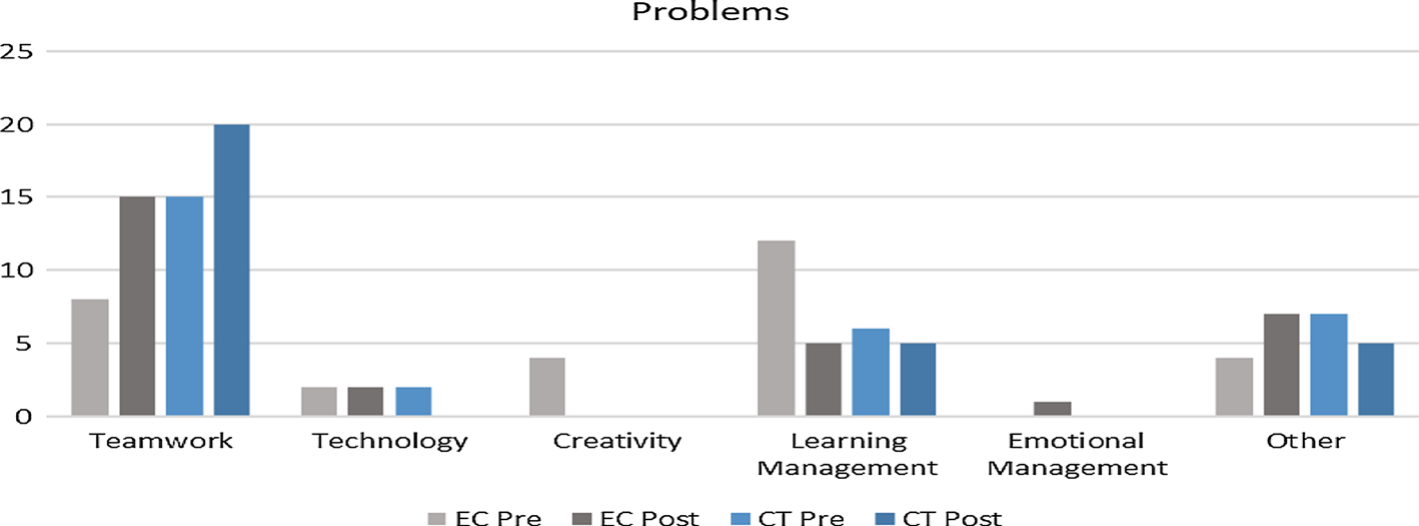
Graphical representation of issues faced as a team

Based on the analysis, it was found that the main issue faced in both groups were related to teamwork (Fig. 9). The CT group reflected higher teamwork issues than the EC group, and in both groups, these issues escalated during the learning process.

**Fig. 10:**
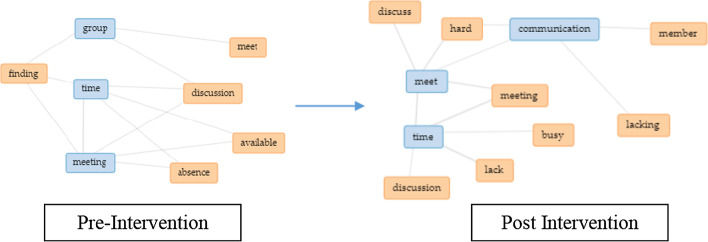
Change in perception for the EC group based on issues faced as a team

**Fig. 11:**
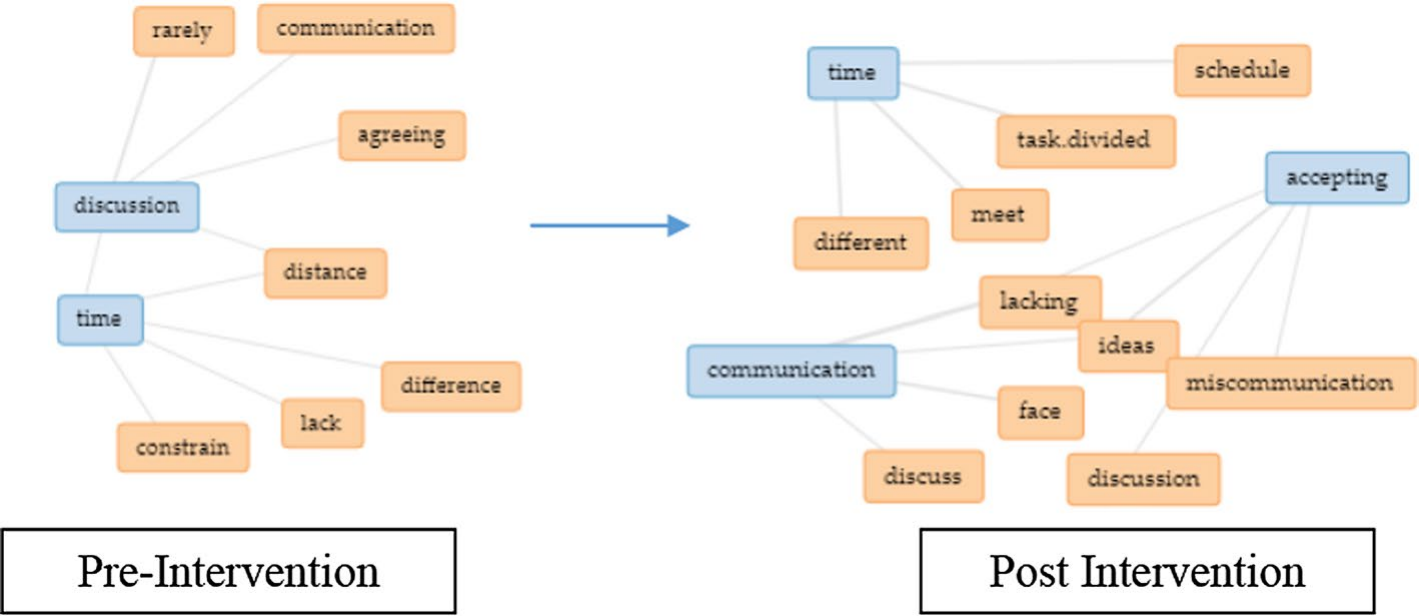
Change in perception for the CT group based on issues faced as a team

Based on analyzing the text, initially, the EC group found issues related to identifying an appropriate time to have group discussions as some teammates were either absent or unavailable (Fig. 10), where a respondent stated that “We can barely meet as a group.” Post-intervention, the group found similar issues, highlighting a lack of communication and availability due to insufficient time and being busy with their learning schedule. Example respond, “We do not have enough time to meet up, and most of us have other work to do.” As for the CT group pre-intervention, similar issues were observed as denoted for the EC group, but communication issues were more prevalent as respondents mentioned differences in opinions or void in feedback which affected how they solved problems collectively (Fig. 11). Example feedback is “One of the members rarely responds in the group discussion.” Post-intervention, the CT group claimed that the main issues besides communication were non-contributing members and bias in task distribution. Examples are “Some of my teammates were not really contributing” and “The task was not distributed fairly.”

iv. Recommendations to improve teamwork

**Fig. 12:**
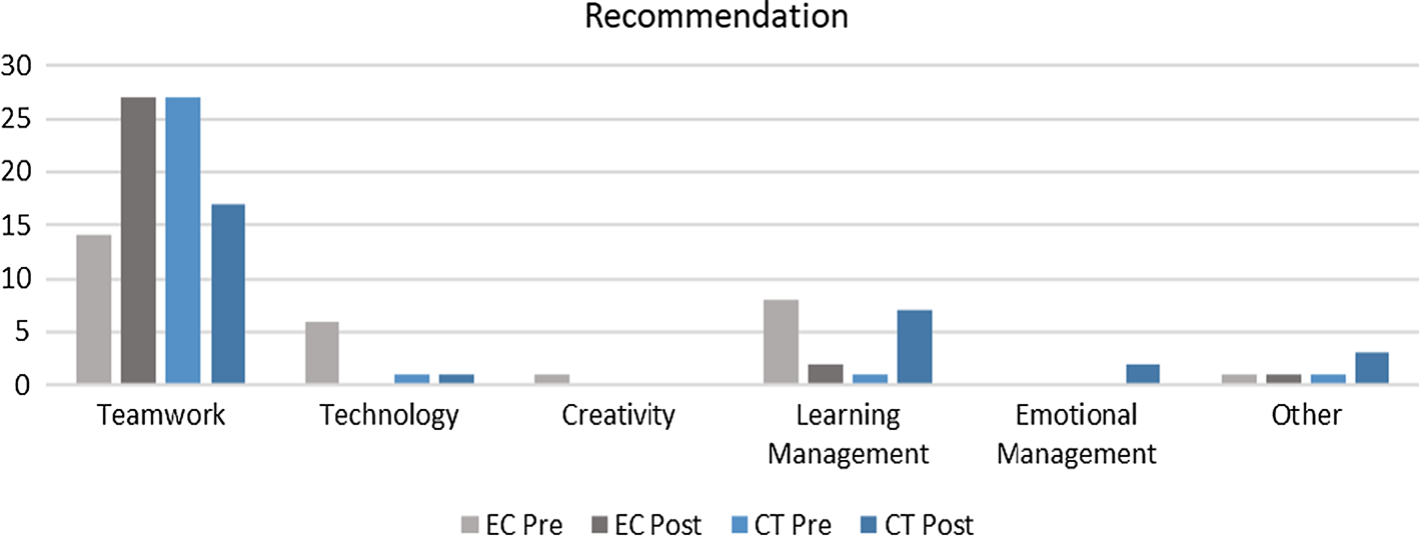
Graphical representation of recommendations pre and post-intervention for both groups

**Fig. 13:**
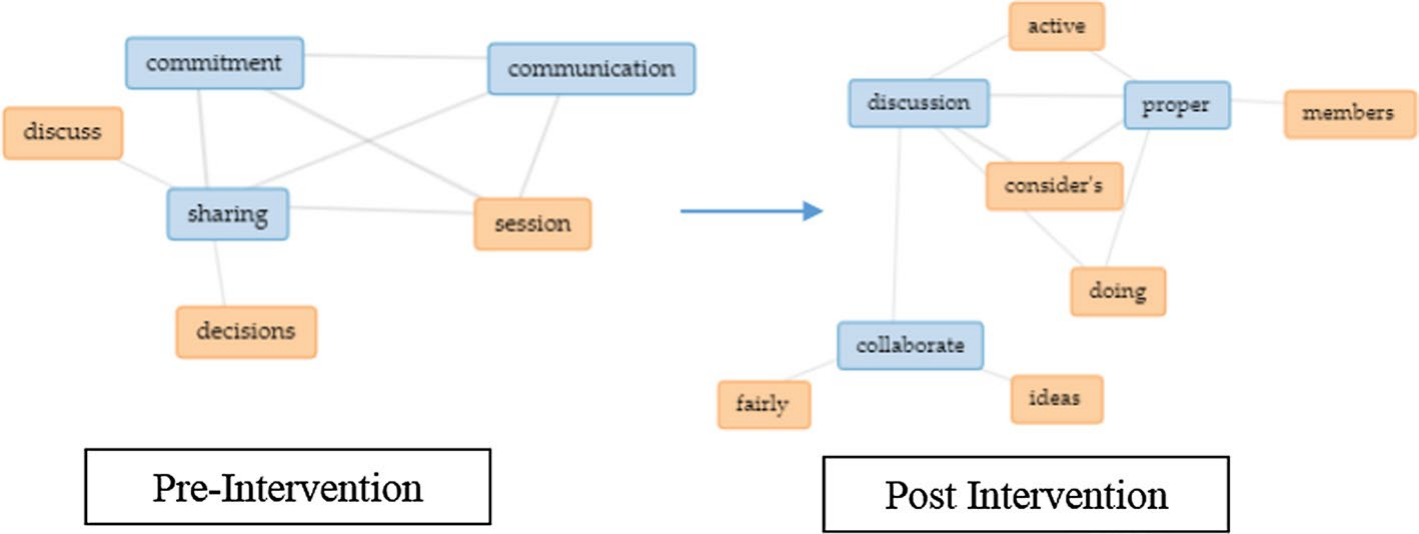
Changes in perception for the EC group based on recommendations for learning improvement as a team

**Fig. 14:**
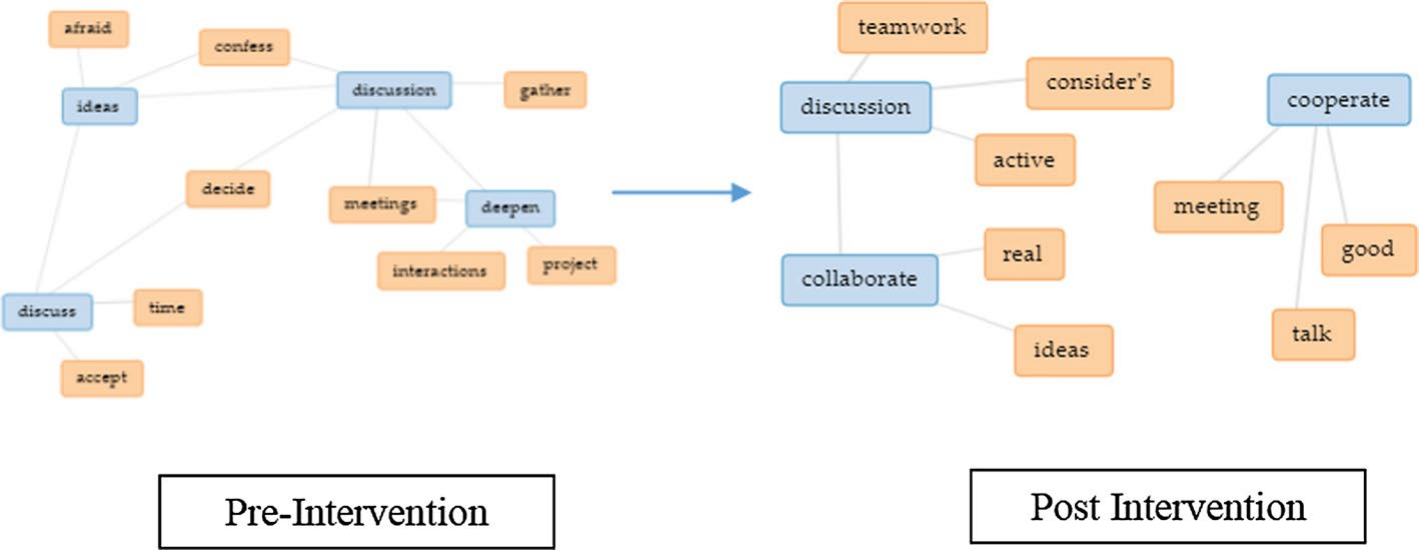
Changes in perception for the CT group based on recommendations for learning improvement as a team

Two interesting trends were observed from Fig. 12, which are (a) EC group reflected more need teamwork whereas the CT group showed otherwise (b) CT group emphasized learning management for teamwork whereas the EC group showed otherwise. When assessing the changes in the EC group (Fig. 13), transformations were observed between pre and post-intervention, where students opined the need for more active collaboration in providing ideas and acceptance. One respondent from the treatment group reflected that acceptance is vital for successful collaboration, stating that “Teamwork and acceptance in a group are important.” Next, for the CT group (Fig. 14), the complexity of defining teamwork pre-intervention, such as communicating, confidence, and contribution of ideas, was transformed to reflect more need for commitment by stating, “Make sure everyone is committed and available to contribute accordingly.”

## Discussion

According to Winkler and Söllner (2018), ECs have the potential to improve learning outcomes due to their ability to personalize the learning experience. This study aims to evaluate the difference in learning outcomes based on the impact of EC on a project-based learning activity. The outcomes were compared quantitively and qualitatively to explore how the introduction of EC will influence learning performance, need for cognition, motivational belief, creative self-efficacy, perception of learning, and teamwork. Based on the findings, EC has influenced learning performance (*d* = 0.978) and teamwork (*d* = 0.641), and based on the Cohen’s *d* value being above 0.42, a significant impact on the outcome was deduced. However, other outcomes such as the need for cognition, motivational belief, creative self-efficacy, and perception of learning did not reflect significant differences between both groups.

Firstly, Kearney et al. (2009) explained that in homogenous teams (as investigated in this study), the need for cognition might have a limited amount of influence as both groups are required to be innovative simultaneously in providing project solutions. Lapina (2020) added that problem-based learning and solving complex problems could improve the need for cognition. Hence, when both classes had the same team-based project task, the homogenous nature of the sampling may have attributed to the similarities in the outcome that overshadowed the effect of the ECs. Equally, for motivational belief, which is the central aspect needed to encourage strategic learning behavior (Yen, 2018). A positive relation with cognitive engagement, performance, and the use of metacognitive strategies (Pintrich & de Groot, 1990) is accredited to the need to regulate and monitor learning (Yilmaz & Baydas, 2017), especially for project-based learning activities (Sart, 2014). Therefore, in both groups, due to the same learning task, these attributes are apparent for both groups as they were able to complete their task (cognitive engagement), and to do so, they were required to plan their task, schedule teamwork activities (metacognition), and design and develop their product systematically.

Moreover, individual personality traits such as motivation have also been found to influence creativity (van Knippenberg & Hirst, 2020) which indirectly influenced the need for cognition (Pan et al., 2020). Nevertheless, these nonsignificant findings may have some interesting contribution as it implies that project-based learning tends to improve these personality-based learning outcomes. At the same time, the introduction of ECs did not create cognitive barriers that would have affected the cognition, motivational and creative processes involved in project-based learning. Furthermore, as there is a triangulated relationship between these outcomes, the author speculates that these outcomes were justified, especially with the small sample size used, as Rosenstein (2019) explained.

However, when EC is reflected as a human-like conversational agent (Ischen et al., 2020) used as a digital assistant in managing and monitoring students (Brindha et al., 2019), the question arises on how do we measure such implication and confirm its capabilities in transforming learning? As a digital assistant, the EC was designed to aid in managing the team-based project where it was intended to communicate with students to inquire about challenges and provide support and guidance in completing their tasks. According to Cunningham-Nelson et al. (2019), such a role improves academic performance as students prioritize such needs. Conversely, for teamwork, technology-mediated communication, such as in ECs, has been found to encourage interaction in team projects (Colace et al., 2018) as they perceived the ECs as helping them to learn more, even when they have communication issues (Fryer et al., 2019). Therefore, supporting the outcome of this study that observed that the EC groups learning performance and teamwork outcome had a more significant effect size than the CT group.

As for the qualitative findings, firstly, even though the perception of learning did not show much variation statistically, the EC group showed additional weightage that implicates group activities, online feedback, and interaction with the lecturer as impactful. Interestingly, the percentage of students that found “interaction with lecturer” and “online feedback and guidance” for the EC was higher than the control group, and this may be reflected as a tendency to perceive the chatbot as an embodiment of the lecturer. Furthermore, as for constructive feedback, the outcomes for both groups were very similar as the critiques were mainly from the teammates and the instructor, and the ECs were not designed to critique the project task.

Next, it was interesting to observe the differences and the similarities in both groups for teamwork. In the EC group, there were changes in terms of how students identified learning from other individual team members towards a collective perspective of learning from the team. Similarly, there was also more emphasis on how they contributed as a team, especially in providing technical support. As for CT, not much difference were observed pre and post-intervention for teamwork; however, the post-intervention in both groups reflected a reduced need for creativity and emphasizing the importance of managing their learning task cognitively and emotionally as a team. Concurrently, it was evident that the self-realization of their value as a contributing team member in both groups increased from pre-intervention to post-intervention, which was higher for the CT group.

Furthermore, in regard to problems faced, it was observed that in the EC group, the perception transformed from collaboration issues towards communicative issues, whereas it was the opposite for the CT group. According to Kumar et al. (2021), collaborative learning has a symbiotic relationship with communication skills in project-based learning. This study identifies a need for more active collaboration in the EC group and commitment for the CT group. Overall, it can be observed that the group task performed through ECs contributed towards team building and collaboration, whereas for the CT group, the concept of individuality was more apparent. Interestingly, no feedback from the EC group mentioned difficulties in using the EC nor complexity in interacting with it. It was presumed that students welcomed such interaction as it provided learning support and understood its significance.

Furthermore, the feedbacks also justified why other variables such as the need for cognition, perception of learning, creativity, self-efficacy, and motivational belief did not show significant differences. For instance, both groups portrayed high self-realization of their value as a team member at the end of the course, and it was deduced that their motivational belief was influenced by higher self-efficacy and intrinsic value. Next, in both groups, creativity was overshadowed by post-intervention teamwork significance. Therefore, we conclude that ECs significantly impact learning performance and teamwork, but affective-motivational improvement may be overshadowed by the homogenous learning process for both groups. Furthermore, it can be perceived that the main contribution of the ECs was creating a “team spirit” especially in completing administrative tasks, interactions, and providing feedback on team progress, and such interaction was fundamental in influencing their learning performance.

### Theoretical and practical implication

This study report theoretical and practical contributions in the area of educational chatbots. Firstly, given the novelty of chatbots in educational research, this study enriched the current body of knowledge and literature in EC design characteristics and impact on learning outcomes. Even though the findings are not practically satisfactory with positive outcomes regarding the affective-motivational learning outcomes, ECs as tutor support did facilitate teamwork and cognitive outcomes that support project-based learning in design education. In view of that, it is worth noting that the embodiment of ECs as a learning assistant does create openness in interaction and interpersonal relationships among peers, especially if the task were designed to facilitate these interactions.

### Limitation and future studies

This study focuses on using chatbots as a learning assistant from an educational perspective by comparing the educational implications with a traditional classroom. Therefore, the outcomes of this study reflected only on the pedagogical outcomes intended for design education and project-based learning and not the interaction behaviors. Even though empirical studies have stipulated the role of chatbots in facilitating learning as a communicative agent, nevertheless instructional designers should consider the underdeveloped role of an intelligent tutoring chatbot (Fryer et al., 2019) and question its limits in an authentic learning environment. As users, the students may have different or higher expectations of EC, which are potentially a spillover from use behavior from chatbots from different service industries. Moreover, questions to ponder are the ethical implication of using EC, especially out of the learning scheduled time, and if such practices are welcomed, warranted, and accepted by today's learner as a much-needed learning strategy. According to Garcia Brustenga et al. (2018), while ECs can perform some administrative tasks and appear more appealing with multimodal strategies, the author questions how successful such strategies will be as a personalized learning environment without the teacher as the EC’s instructional designer. Therefore, future studies should look into educators' challenges, needs, and competencies and align them in fulfill EC facilitated learning goals. Furthermore, there is much to be explored in understanding the complex dynamics of human–computer interaction in realizing such a goal, especially educational goals that are currently being influenced by the onset of the Covid-19 pandemic. Conversely, future studies should look into different learning outcomes, social media use, personality, age, culture, context, and use behavior to understand the use of chatbots for education.

## Data Availability

The data that support the findings of this study are available from the corresponding author upon reasonable request.

## Abbreviations

EC: Educational chatbots
CT: Control group
R*i*pe: Reliability, interpersonal communication, pedagogy, and experience
GORE: Goal-oriented requirements engineering
